# A Single-Center Study Evaluating the Effects of a Novel Retinol and Cannabidiol Combination Topical on Facial Skin

**DOI:** 10.1093/asjof/ojac002

**Published:** 2022-01-27

**Authors:** Julius Few, Michael J Lee, Alec Semersky, Emily Mariscal, Ginny Vachon

## Abstract

**Background:**

While retinol is known to reduce the appearance of fine lines and wrinkles, it is associated with irritating effects. However, when combined with water soluble cannabidiol (CBD; CR Topical), CBD may act to reduce oxidative stress and inflammation, mitigating irritation from retinol and further improving the skin’s appearance through independent anti-aging mechanisms.

**Objectives:**

To assess the efficacy and tolerability of CR-Topical for improving facial skin.

**Methods:**

In this prospective, single-center pilot study, 9 female patients and one male patient aged 20 to 53 years who presented with facial skin imperfections (visible pores, dehydration, roughness, and/or static/dynamic wrinkles) applied CR-Topical to the entire face once daily for 42 days. Outcomes were measured on days 1, 21, and 42 using the Global Ranking Scale (GRS) with Comprehensive Skin Analysis by the patient and senior investigator as well as by a blinded reviewer (board-certified plastic surgeon). Dynamic videos and static imagery were taken before and after treatment, and patient satisfaction surveys were completed.

**Results:**

Global improvement across all 13 domains was observed, with the greatest mean differences for visible pores (2.0; 95% CI, 1.5-2.5), dehydration (2.0; 95% CI, 1.4-2.6), surface roughness (1.8; 95% CI, 1.2-2.4), static wrinkles (1.8; 95% CI, 1.1-2.5), and dynamic wrinkles (1.6; 95% CI, 0.8-2.3). Patient satisfaction (100%) and willingness to recommend the product to others (90%) were high, and tolerability of CR-Topical was excellent.

**Conclusions:**

CR-Topical is effective at improving global skin quality, including static and dynamic wrinkles. This study also used 4-dimensional analysis in the evaluation, a novel and developing method.

**Level of Evidence: 4:**

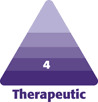

Skin aging is influenced by a number of factors, including extrinsic factors, such as exposure to ultraviolet (UV) light, poor diet, or pollutants, and intrinsic factors, such as collagen and elastin loss over time.^1^ Within the skin, intrinsic aging is due, in part, to the decreased capacity of keratinocytes, fibroblasts, and melanocytes to proliferate and the degeneration of the fibrous extracellular matrix, whereas 80% of extrinsic aging can be attributed to UV radiation.^2-4^ Interestingly, for both intrinsic and extrinsic aging, reactive oxygen species (ROS) and associated oxidative stress can accelerate these processes in the skin through multiple mechanisms and contribute to dyspigmentation, reduced barrier function, laxity, and uneven texture.^1,5^ In addition, ROS are associated with inflammation, which can further negatively impact the skin’s appearance.^5,6^

In clinical practice, one of the few topical treatments for which there is strong clinical evidence of efficacy for reducing the signs of aging is retinol.^7^ The anti-aging effects of retinol are manifested by promoting proliferation of keratinocytes, strengthening the epidermis, and increasing collagen; however, retinol may indirectly decrease the antioxidative effects of the skin, leaving it prone to damage from ROS.^8-10^ Furthermore, skin reactions such as redness, drying, peeling, or burning associated with retinol treatment can lead to inconsistent use or discontinuation by patients. In the current study, purified, water-soluble cannabidiol (CBD) was evaluated with 0.2% retinol as a combination product (CR-Topical, Aforé, Chicago, IL). By combining these agents, CR-Topical face cream was designed to combat both the intrinsic and extrinsic factors involved in skin aging by increasing skin turnover while protecting the skin through antioxidative and anti-inflammatory mechanisms, such that both efficacy and tolerability are improved.

In the current pilot study, due to the novelty of the combination and the need to characterize the impact of these treatments on the skin, a more granular approach was taken to measuring outcomes. Typically, aesthetic conditions are measured using validated and non-validated scales (eg, FACE-Q and Global Aesthetic Improvement Scale) and using static photographs taken from multiple angles.^11,12^ Patient satisfaction questionnaires may also be used to gauge perceptions of efficacy, satisfaction, and overall impressions. However, these scales are limited in their ability to detect specific changes to features such as skin quality. Therefore, the 9-domain Global Ranking Scale (GRS) and 4-domain Skin Quality assessment were applied.^13^ The GRS is unique in that it is used as a collaborative tool by the physician with the patient, and therefore additional methods, described later, were used to confirm observations, including blinded review and ordering of before and after images by a board-certified plastic surgeon and inclusion of patient survey questions, which were completed independently, that overlap with GRS skin quality domains to confirm outcomes.

In addition to the capture of static images, this study evaluated patients using video of a series of facial expressions as an exploratory endpoint. Even the best before and after static images represent an incomplete picture of treatment efficacy. Moreover, optimal static correction can result in unnatural mimetic movement, overfilling, and/or inadequate or imbalanced correction. Compared with static images, dynamic videos are better able to capture global improvement across 4 dimensions, encompassing glide plane movement and multiple facial expressions (**Table 1**). The use of videos in this study was exploratory in nature and represents the first time that this 4-dimensional beauty (4D Beauty, The Few Institute, Chicago IL) assessment has been used in a clinical study. So much of how patients perceive themselves and are perceived by others is embedded in expressions and even qualities such as facial positioning and openness can be appreciated with video. This type of assessment is in its infancy, and development will likely involve extensive multidisciplinary research and development of novel measures, as well as protocols for video capture. In this pilot study, the use of 4D Beauty assessment is detailed but is not part of the formal evaluation of efficacy. However, this work paves the way for use in clinical practice and study.^13,14^

**Table 1.** Four-Dimensional vs Static Measurement of Facial Skin Improvement

Four dimensions	Static dimensions
• Natural-looking facial expressions and transitions between facial expressions (glide planes) • Changes apparent during communication • Consistent age-appropriate correction across the face	• Single-expression • Noncommunicative changes in appearance • Limited measure of “rejuvenation” • Can miss imbalances or overcorrection and/or give the impression of suboptimal correction

## METHODS

This single-center pilot study evaluated the efficacy, safety, and tolerability of CR-Topical (300 mg of purified, medical-grade, water-soluble CBD; 0.2% retinol; proprietary botanicals; and stabilizing agents) applied once daily to the facial skin. The study enrolled 10 healthy (9 female and 1 male) patients between 20 and 53 years of age who presented with any combination of the following on the facial skin: visible pores, dehydration, roughness, static or dynamic wrinkles, skin laxity, and/or facial fine lines. Patients were informed of the potential for exposure to CBD and were blinded as to whether they were receiving active treatment or a placebo. Each patient was provided with a tube of CR-Topical labeled “A” by the manufacturer and instructed to apply 2 pumps (0.3 mL) of CR-Topical to their entire facial skin in the evening before bed after using a mild facial cleanser. After enrollment, patients immediately began treatment and continued the regimen for 42 days (6 weeks) until study completion. For the duration of the study, patients were asked to fill out a daily log detailing any changes to their skin appearance and any side effects they experienced. Patients served as their own control.

Patients who had botulinum toxin or filler within 3 months of study enrollment were excluded. Additionally, patients who used topical, inhaled, or ingested cannabis and/or hemp-derived products including CBD or tetrahydrocannabinol within 30 days of the study start date or during the study period were excluded. Further key exclusionary characteristics included facial rejuvenation procedures (eg, chemical peels, microneedling, and microdermabrasion), pregnancy, advanced or poorly controlled diabetes, current smoker or history of heavy smoking, the use of anti-inflammatory topical products during the study period, or regular continuous use of systemic or topical corticosteroids on the area to be treated.

**Figure 1.**
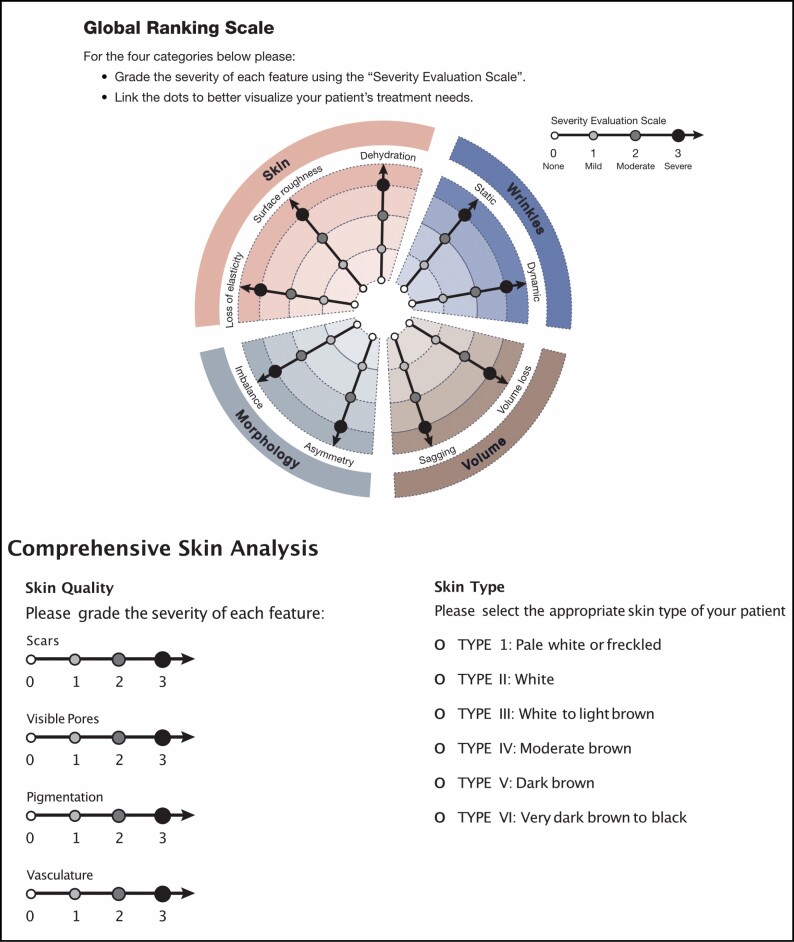
Global Ranking Scale and Comprehensive Skin Analysis. Scale adapted from Jain et al.^13^.

The primary efficacy endpoint consisted of clinical evaluations of the face based on comparison of GRS scores at each time point, compared with baseline, as well as qualitative comparison of standardized before and after photographs on days 1, 21, and 42. During visits on days 1 (baseline), 21, and 42, standardized photographs of the treatment areas were taken, and global skin appearance was assessed using the GRS with Comprehensive Skin Analysis by joint evaluation of severity by the lead investigator and the patient (**Figure 1**).^13^ Note, the investigator and patient were blinded to their original assessment scores at the time of evaluation. Each of the scale’s 13 domains, including loss of elasticity, surface roughness, dehydration, static and dynamic wrinkles, volume loss, sagging, asymmetry, imbalance, scar presence, visible pores, pigmentation, and vasculature, was graded from 0 to 3 (0, none; 1, mild; 2, moderate; and 3, severe), and mean scores at baseline and at 42 days were calculated for each domain and compared.^13^ Paired mean difference with each patient used as their own control, and the lower and upper 95% CIs for each domain were calculated. Due to the small number of patients and the use of a 4-point scale across numerous domains, paired *t*-tests with *P*-values are less informative (*P*-values calculated using a paired 2-tailed *t*-test were significant for all measures assessed) and are not presented here.

Because the GRS is intended to be used as a collaborative tool by the physician with the patient, it is not conducive to blinded review. Instead, a blinded reviewer was asked to order the static baseline and 42-day images as before and after, and percent agreement with whether the actual photograph was taken at baseline or Day 42 was calculated. In addition, questions overlapping with GRS skin quality domains were included in the patient questionnaire to confirm outcomes. These questionnaires were completed by patients independently, following completion of the GRS.

Secondary efficacy endpoints included patient observations from satisfaction questionnaires regarding major cutaneous changes such as smoothness, irritation, pruritus, burning, and erythema. Exploratory endpoints included the use of 4D assessment as a tool for a qualitative measure of dynamic and animation-related patient features. On days 21 and 42, daily patient diaries were reviewed for compliance and tolerability, and patients completed an 8-question satisfaction survey. Filmed video evaluations occurred on days 1 and 42 for 4D assessment.

Safety was monitored by the investigator through application site assessment during each follow-up visit (days 1, 21, and 42). Because of the established propensity for retinol to cause irritation, an additional, virtual safety assessment of the application site occurred on Day 3. At each follow-up visit, study medication tubes were weighed for compliance. This study was approved by an institutional review board (Advarra IRB, Columbia, MD) and adhered to the Good Clinical Practice and standards outlined in the World Medical Association’s Declaration of Helsinki. Written consent was provided, by which the patients agreed to the use and analysis of their data.

## RESULTS

Ten patients were enrolled in this pilot study, with a median age of 42.5 (**Table 2**). A greater proportion of patients had either white (30%) or white to light brown (30%) skin compared with other skin types, as determined using the GRS scale.^13^ All 10 patients reported using CR-Topical once daily at night before bed for 42 days and product weight was consistent with the high level of compliance reported. No patients were lost to follow-up.

**Table 2.** Subject Characteristics

Subject characteristics (N = 10)	
*Characteristic*	*Value*
Mean age, years (range)	42.5 (20-53)
GRS skin type, n (%)	
I: pale white or freckled	1 (10)
II: white	3 (30)
III: white to light brown	3 (30)
IV: moderate brown	1 (10)
V: dark brown	2 (20)
VI: very dark brown to black	0 (0)

GRS, Global Ranking Scale.

**Figure 2.**
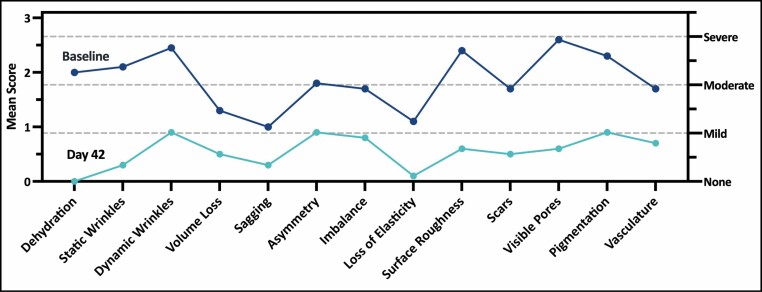
Facial skin appearance across domains at baseline (blue) and Day 42 (teal).

At 42 days, GRS with Comprehensive Skin Analysis score improvement was observed from baseline to Day 42 across all 13 domains, including domains for which improvement was unexpected (eg, sagging). While improvement was consistent, some areas improved more than others, generally those most consistent with a topical treatment (**Figure 2**). Among patients, the highest mean scores at baseline were visible pores (2.6), dynamic wrinkles (2.5), surface roughness (2.4), pigmentation (2.3), and static wrinkles (2; higher mean scores correlate to worse skin quality [0, none; 1, mild; 2, moderate; and 3, severe]).

**Figure 3.**
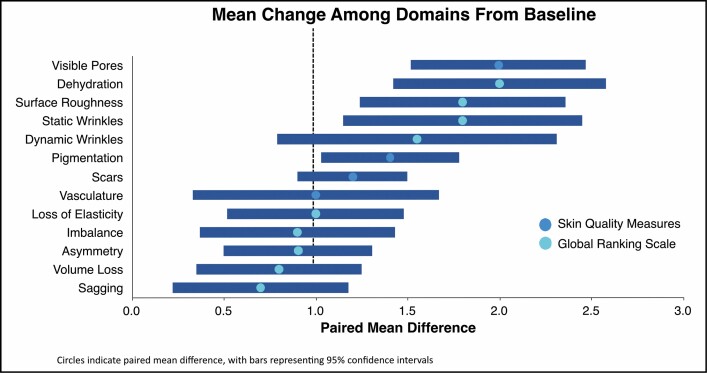
Mean change among domains from baseline for the Global Ranking Scale (teal circles) and Skin Quality domains (blue circles) with 95% CIs (navy blue bars).

When assessing the relative degree of improvement for each domain, those with ≥ 1-point improvement from Day 0 to 42 were found to be consistent with topical treatments (**Figure 3**, black dotted line). While statistically significant changes were observed for each domain including both the GRS and skin quality measures, the areas of greatest change were visible pores (2.0-point change; 95% CI, 1.5-2.5), dehydration (2.0-point change; 95% CI, 1.4-2.6), surface roughness (1.8-point change; 95% CI, 1.2-2.4), static wrinkles (1.8-point change; 95% CI, 1.1-2.5), and dynamic wrinkles (1.6-point change; 95% CI, 0.8-2.3). Notably, while the greatest possible score change is 3, not all patients began with “severe” skin quality, making a 3-point change impossible. However, a 2-point change represents a change from “severe” to “mild” or “moderate” to “none,” both of which represent very impactful improvements. Each patient who reported severe static wrinkles, dynamic wrinkles, surface roughness, scars, visible pores, or vasculature at baseline reported at least “mild” severity for these domains at study completion. Moreover, all patients with “severe” dehydration reported “none” at the end of the study. The blinded reviewer correctly ordered before and after images for 80% of the patients.

**Figure 4.**
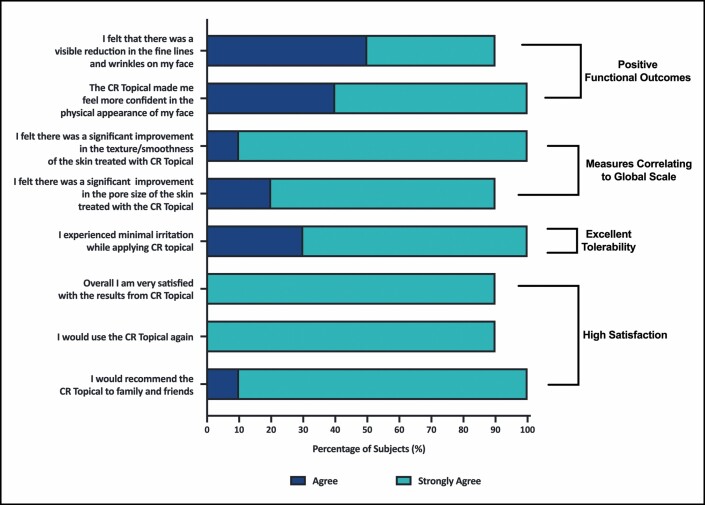
Patient satisfaction and functional outcomes measured using the patient survey.

Satisfaction and functional outcomes assessed using patient questionnaires completed on days 21 and 42 revealed that on Day 42, 90% of patients either agreed (50%) or strongly agreed (40%) that they experienced a visible reduction of facial fine lines and wrinkles (**Figure 4**). Overall, 100% of patients felt that CR-Topical made them more confident in the physical appearance of their face. In agreement with GRS outcomes, patients strongly agreed that the use of CR-Topical significantly improved the texture/smoothness (90%) and pore size (70%) of the skin, outcomes consistent with those measured with the GRS. Patients either agreed or strongly agreed (30% vs 70%, respectively) with the statement that they experienced minimal irritation for the duration of the study, demonstrating the excellent tolerability of CR-Topical. Furthermore, 90% of patients strongly agreed that they were very satisfied with the product, with 90% expressing both their willingness to use CR-Topical again and recommend the cream to family and friends.

**Figure 5.**
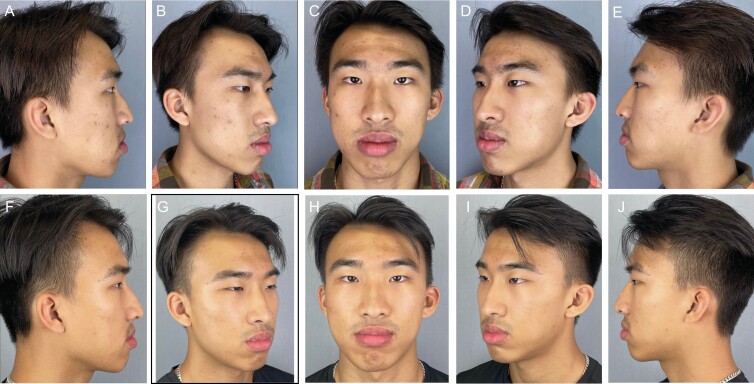
A 20-year-old male (A-E) at baseline with facial skin characterized by active acne blemishes, acne scarring, and hyperpigmentation; and (F-J) after 42 days of nightly application of CR-Topical. Note the significant decrease in the intensity of acne-related hyperpigmentation and the appearance of inflammation. Additionally, the facial skin is more hydrated, indicated by the more even distribution of light.

**Figure 6.**
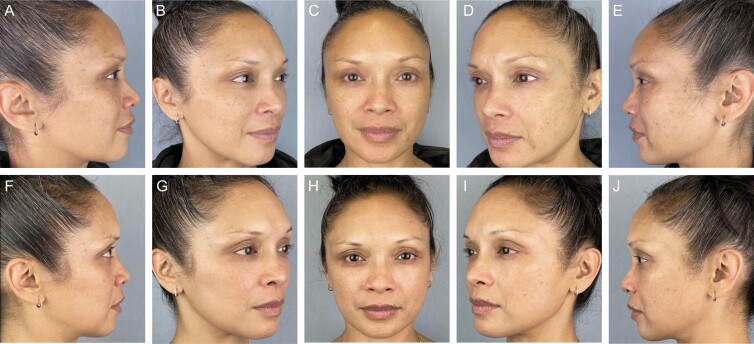
A 53-year-old female (A-E) at baseline with facial skin characterized by excessive global fine lines and lentigines; and (F-J) after 42 days of nightly application of CR-Topical. Note the shallow depths of the fine wrinkles in the running vertically on the cheek and horizontally in the crow’s feet region. Additionally, the highly pigmented lentigines brought on by excessive sun or age have lightened significantly.

**Figure 7.**
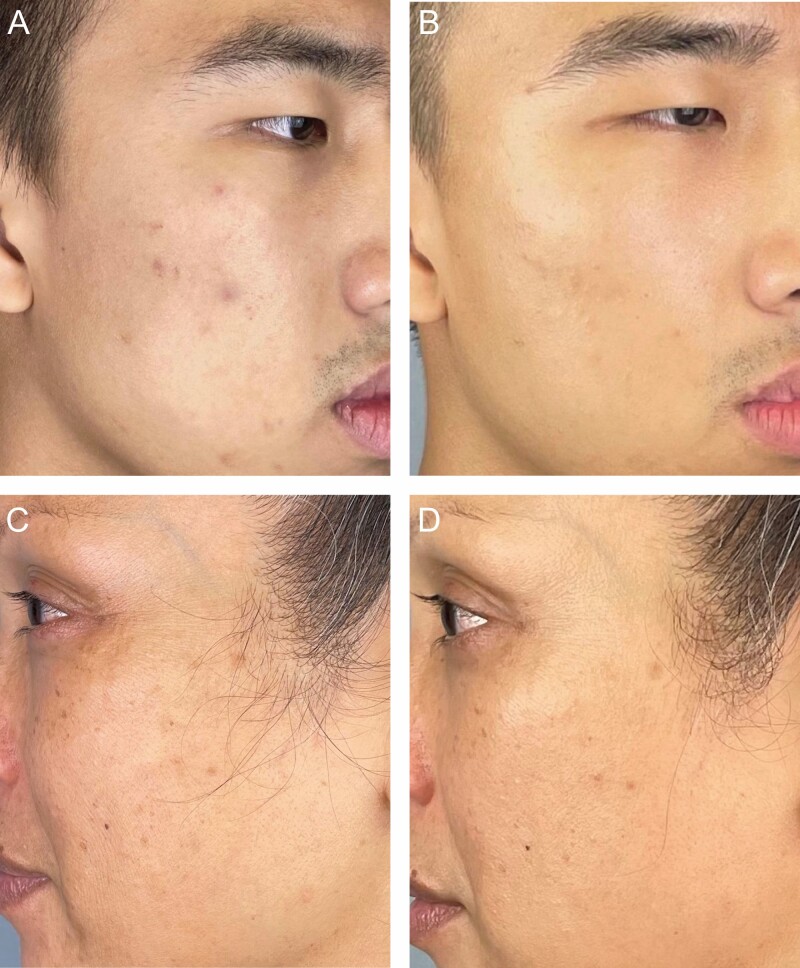
Representative close up images of the 20-year-old male shown in Figure 5 (A) at baseline and (B) after 42 days of nightly applications of CR Topical; and the 53-year-old female shown in Figure 6 (C) at baseline and (D) after 42 days of nightly application of CR Topical.

Representative patient images (**Figures 5, 6**) illustrate improvement across a range of GRS domains. Changes in the skin quality are apparent in before and after images, and **Figure 7** shows a close-up view of each patient so that changes in skin quality can be appreciated.

## DISCUSSION

This pilot study demonstrated that CR-Topical, a novel formulation of retinol, peptides, and antioxidants combined with water-soluble CBD, increases global skin quality and leads to positive patient functional outcomes. While retinol is correlated to a decrease in the depth of fine lines and wrinkles, it is known to have irritating effects on the skin that can lead to erythema, pruritus, peeling, and redness with long-term use.^15^ Here, these negative effects appear to be are counteracted by CBD, improving the tolerability of the product. It will be important to characterize relative tolerability in a split-face study in the future.

Although research on CBD oil as a topical agent is still emerging, supplementary use has been shown to decrease inflammation and improve therapeutic outcomes for severe inflammatory skin diseases, supporting this potential role.^16-18^ Anecdotally, the improvements observed for the combination of retinol and water-soluble CBD are better than for either product alone, suggesting an additive, or potentially synergistic benefit. Larger controlled studies will need to be carried out in order to understand the relative effects of CR-Topical and retinol and to define any synergistic activity for the 2 ingredients. Importantly in these studies, a larger number of patients should be followed for an extended period of time to best understand the impact of treatment.

When considering the potential for synergistic activity, the mechanism of action for broth retinol and CBD is informative. Through the combined effects of promoting cell proliferation and reducing oxidative stress, these agents may work synergistically to combat both the extrinsic and intrinsic causes of aging. While the mechanism of action of CBD in the skin has yet to be fully elucidated, its antioxidant and anti-inflammatory activities are well recognized and can be partially attributed to its activation of Nrf2-target activation genes, including *HMOX1*, which plays crucial roles in modulating inflammation, apoptosis, and oxidative stress.^19^ Importantly, CBD-induced activation of *HMOX1* expression occurs through an Nrf2-independent mechanism.^19^ Because retinol suppresses the expression of Nrf2-target antioxidative genes, the addition of CBD oil can potentially restore antioxidative effects inhibited by retinol while allowing for retention of skin-strengthening properties.^8,10,18^ This activity of CBD is in addition to independent mechanisms, including modulation of the human endocannabinoid system, which is linked to anti-aging effects through its role in maintaining skin homeostasis and barrier function, antioxidant and anti-inflammatory activity, as well optimal sebum production.^18-20^ For Retinol, independent effects are manifested through promoting keratinocyte proliferation, strengthening of the epidermis, and increasing collagen (through the protection of existing collagen and stimulation of neocollagenesis) in the skin.^8,9^ Importantly, the combination of CBD and retinol appears to reduce skin irritation often observed with retinol alone. Notably, 100% of patients who reported “severe” dehydration at baseline reported “none” at the conclusion of the study, indicating that CBD may combat the irritation characteristic of retinol topicals, providing considerable benefit to patient tolerability and indirect improvement in efficacy due to increasing the likelihood of adherence. Importantly, the CBD used in this study is water soluble. Most often, CBD is administered dissolved in oil, commonly coconut oil or medium-chain triglyceride oil. The absence of oils in this formulation that could potentially clog pores or otherwise irritate the skin is also an important contributor.

While improvement across all 13 domains for all patients suggests a global effect of CR-Topical, improvements in areas such as sagging or asymmetry may signal some variability in day-to-day perceptions, some level of bias that stems from the investigator and the patient completing the assessment together or the incomplete ability of the patient to differentiate entirely between the contributions of individual domains to overall global improvement. In future studies, placebo control and blinding both the patient and evaluating investigator will shed light on this topic. Additionally, when interpreting results, it is important to consider that the rating scale ranged from 0 to 3. Therefore, seemingly modest changes (eg, “severe” at 3 to “mild” at 1) do indeed reflect a significant improvement. Because of the granular nature of the GRS scale, it is possible that future studies could also evaluate combination treatment with other products or modalities vs monotherapy, further characterizing the effects of distinct interventions on global outcomes when used together.

As skin treatments continue to emerge and become increasingly multi-modal in nature, it is essential to better understand the specific effects that different treatments have on the skin. While historical measurements for skin treatment efficacy have relied on static imagery, this methodology cannot capture the movement of glide planes and facial expressions, and it is how our faces move during communication that most informs perceptions of beauty. Capturing movements of the face with short videos more accurately reflects what is observed in day-to-day life as opposed to static imagery and provides a real-world context for evaluating the most relevant effects of treatment. This novel and dynamic 4D assessment may prevent overcorrection or unnatural-looking results by encouraging consideration of youthful dynamics in expression as well as facial proportion.^21^ Furthermore, 4D assessment can be used in conjunction with new or existing scales and opens the door for incorporation of new measures rating naturalness of outcomes, projection or correction across multiple facial expressions, apparent confidence, openness, or other characteristics that are not traditionally thought of as study outcomes. Moreover, patients can use the videos to better evaluate their overall satisfaction and experience by observing their facial movements before and after treatment. Like the GRS, 4D assessment can be used as a tool for patient education, treatment planning, and encouraging pursuit of natural-looking results. The exploratory assessment using 4D dynamic imagery in this study revealed that video documentation and 4D beauty are important elements of a comprehensive efficacy profile for any product or device and paved the way for video and dynamic assessments as measures of treatment effect. 

Shortcomings of this study include the small sample size and short duration of follow-up time. A larger cohort and increased length of the study could lead to more robust results, especially if the design includes a control group or is a split-face study. Future studies will compare the efficacy of CR-Topical with retinol alone.

## CONCLUSIONS

When used in combination, water-soluble CBD and retinol offer significant improvements in skin quality, as well as in static and dynamic lines, with minimal irritation. The synergistic activity of CBD and retinol may explain this high level of efficacy. Additionally, a more granular assessment of aesthetic outcomes is long overdue and should be incorporated into future clinical studies evaluating topical skin treatments. Dynamic, 4D beauty assessment is a newly emerging mode of evaluation that could be fully developed and applied to better capture the effects of treatment.
